# Antifungal Activity of Bioactive Compounds Produced by the Endophytic Fungus *Paecilomyces sp*. (JN227071.1) against *Rhizoctonia solani*

**DOI:** 10.1155/2023/2411555

**Published:** 2023-04-20

**Authors:** Sumaiya Naeema Hawar, Zainab K. Taha, Atyaf Saied Hamied, Hanady S. Al-Shmgani, Ghassan M. Sulaiman, Sobhy E. Elsilk

**Affiliations:** ^1^Biology Department, College of Education for Pure Science, Ibn Al-Haitham, University of Baghdad, Baghdad, Iraq; ^2^Ministry of Education, First Resafa Education Directorate, Al-Mutamizat High School for Girls, Baghdad, Iraq; ^3^Division of Biotechnology, Department of Applied Sciences, University of Technology, Baghdad, Iraq; ^4^Botany and Microbiology Department, Faculty of Science, Tanta University, Tanta 31527, Egypt

## Abstract

Biologically active natural compounds are molecules produced by plants or plant-related microbes, such as endophytes. Many of these metabolites have a wide range of antimicrobial activities and other pharmaceutical properties. This study aimed to evaluate (*in vitro*) the antifungal activities of the secondary metabolites obtained from *Paecilomyces sp.* against the pathogenic fungus *Rhizoctonia solani*. The endophytic fungus *Paecilomyces* was isolated from *Moringa oleifera* leaves and cultured on potato dextrose broth for the production of the fungal metabolites. The activity of *Paecilomyces* filtrate against the radial growth of *Rhizoctonia solani* was tested by mixing the filtrate with potato dextrose agar medium at concentrations of 15%, 30%, 45%, and 60%, for which the percentages of inhibition of the radial growth were 37.5, 50, 52.5, and 56.25%, respectively. The dual culture method was conducted on PDA medium to observe the antagonistic nature of the antibiotic impacts of *Paecilomyces sp.* towards the pathogenic fungus. The strength of the antagonistic impacts was manifested by a 76.25% inhibition rate, on a scale of 4 antagonistic levels. Ethyl acetate extract of *Paecilomyces sp.* was obtained by liquid-liquid partition of the broth containing the fungus. Gas chromatography-mass spectrometry (GC-MS) analysis identified the presence of important chemical components *e.g.,* (E) 9, cis-13-Octadecenoic acid, methyl ester (48.607), 1-Heptacosanol, 1-Nonadecene, Cyclotetracosane (5.979), 1,2-Benzenedicarboxylic acid, butyl 2-methylpropyl ester, di-sec-butyl phthalate (3.829), 1-Nonadecene, *n*-Nonadecanol-1, Behenic alcohol (3.298), *n*-Heptadecanol-1, 1-hexadecanol, *n*-Pentadecanol (2.962), Dodecanoic acid (2.849), 2,3-Dihydroxypropyl ester, oleic acid, 9-Octadecenal, and (Z)-(2.730). These results suggest that secondary metabolites of the endophytic *Paecilomyces* possess antifungal properties and could potentially be utilized in various applications, such as environmental protection and medicine.

## 1. Introduction

Biologically active natural compounds are molecules that are produced either by plants or plant-related microorganisms, such as endophytes. Many of these metabolites have a wide range of antimicrobial activities and other pharmaceutical properties [1]. Endophytic fungi are microorganisms that are held within plant tissue without stimulating obvious disease symptoms [2]. They are an important source of many secondary metabolites that are biologically active [3], such as tannins, flavonoids, coumarins, alkaloids, phenolic, peptides, lactones, phenylpropanoids, terpenes, polyketides, and lignans [4]. Fungal endophytes are a natural source of new, physiologically active substances that are significant for medicine [5]. Anticancer and antimicrobial agents derived from the endophytes fungi (more than 70%) are biologically active natural compounds or their derivatives [6]. Biologically active compounds demonstrated significant impacts on immunological diseases, such as hypocholesterolaemia and diabetes, as well as issues related to oxidative stress. They are also helpful in enhancing crops and reducing the negative effects of abiotic stresses [7].

*Paecilomyces* is a widespread filamentous fungus that inhabits environments such as food products, soil, and decomposing plants. It includes various species that are both harmful and saprophytic [8], some species can infect humans [9], nematodes [10], and can grow on the rhizosphere of many plants [11]. The interaction between the plant and *Paecilomyces* may also improve plant health and provide protection against plant pathogens through different mechanisms [12]. *Paecilomyces* acts as a biological control agent and therefore has positive effects on crop growth [13]. The genus *Pacecilomyces* has numerous species which can produce different secondary metabolites; with a total of 148 active metabolites have been reported [14] that can be used for agrochemicals or drugs development. They possess diverse biological activities, including cytotoxic [15], fungicidal [16], insecticidal [17], herbicidal [18], bactericidal [19], nematicidal [20], and antitumour [21]. Endophytic fungi help in promote plant competence through several mechanisms of action. The modes of action include endophytes-pathogens interactions (direct mechanisms) such as the production of antibiotics [22] and lytic enzymes [23], even though enzymes may not be merely effective as antagonism agent, they may enhance antagonistic activities when combined with other mechanisms. Competition is a powerful mechanism used by endophytes in preventing pathogens from colonizing the host tissue [24] and enhanced plant defense mechanisms (indirect mechanism) endophytes increase the plant defense mechanism by production of secondary metabolites and enhanced resistance of plant host. An example of innate pathogen resistance that has been developed may be a specific or unspecified resistance [25].

Many crop species are susceptible to the pathogenic fungus *Rhizoctonia solani.* It is the causal organism of stem canker and black scurf in potatoes (*Solanum tuberosum* L.). Being the latter the most prevalent disease in the nation [26]. Disease caused by *Rhizoctonia* can result in marketable yield losses of up to 30% in potato and considerable losses in quality. While quantitative losses result from infections of stems, stolons, and roots, which affect tuber size and number, qualitative losses are mostly caused by the creation of malformed tubers and the growth of sclerotia on the tuber surface (known as black scurf) [27]. Thus, as potatoes that are one of the basic nutrition sources of the world population, the purpose of the current study is to assess the antifungal activity against *Rhizoctonia solani* of secondary metabolites obtained from *Paecilomyces sp.*

## 2. Materials and Methods

### 2.1. Endophytic Fungus

The endophytic fungus *Paecilomyces sp*. (JN227071.1) was previously isolated and identified by polymerase chain reaction technique (PCR) [28] from *Moringa olifera* leaves. The fungal isolate was cultivated on potato dextrose agar (PDA) and stored in refrigerator at 4°C, the culture was transferred out every two months in new PDA medium.

### 2.2. Fungi Testing Culture

The pathogenic fungus *Rhizoctonia solani* was obtained from the Department of plant protection, Agriculture College, University of Baghdad. The fungus was maintained on PDA slant and stored at 4°C, before use transferred to PDA plate for 5–7 days at 28°C.

### 2.3. Fermentation and Extraction

In order to obtain bioactive compounds from *Paecilomyces sp*., the fungus isolate was cultured in potato dextrose broth (PDB). Conical flask (250 ml) containing 100 ml of PDB were inoculated with three agar plugs (5 mm) from 7 day old fungal, incubated at 28°C, 120 rpm for 14 days. Fermentation broth and fungal biomass were separated using a-Whatman filter paper (no. 1), and the culture broth was extracted by ethyl acetate (1 : 1 v\v). The organic extracts were evaporated by oven at (50°C) [29].

### 2.4. Antifungal Activity Assay on Potato Slices

Slices of potato tubers were used to investigate the bioactive chemicals produced by *Paecilomyces Sp.*; specifically for their ability to protect against *Rhizoctonia solani*, according to [30] with modifications. To sterilize the surface of potato tubers, sodium hypochlorite (2%), and (70%) ethanol alcohol were used; they were then washed three times with sterile distilled water (10 minutes each) and cut into slices under sterilized conditions. Potato slices were flooded in the fungal filtration medium or the PDB medium (control solution) for one hour in sterile dishes. The potato slices were then removed from the immersion solution, blotted with sterile filter paper to remove excess solution, and air-dried. They were then put in sterile dishes, while some slices were inoculate with a disk (5 mm) of pathogenic fungi and incubated at 28°C for 7 days. Each treatment was performed with three replicates.

### 2.5. Effects of *Paecilomyces* Filtrate on *Rhizoctonia solani* Growth

The growth of the pathogenic fungus *Rhizoctonia solani* was investigated to assess the effects of *Paecilomyces* filtrate. Fungal filtrate was added after sterilize with Millipore filter (0.22 *μ*m) to the growth medium to obtain concentration of 15%, 30, 45, and 60%. Plates without fungal filtrate served as control. The mixture was mixed well and then poured in petri dishes until hardened. *Rhizoctonia solani* 5 mm disks (7 days old) were placed in the middle of the petri dishes and incubated at 28°C for 7 days. The growth inhibition percentage was calculated according to [31].

### 2.6. Test of Antagonistic Activity

The dual culture-plate antagonism assay was used to assess the influence of the endophytic fungus *Paecilomyces sp.* against *R. solani* [31]. The fungi were grown for seven days at 28°C on PDA medium in PDA Petri dishes. 5-mm plugs of *Paecilomyces sp*. and *R. solani* were co-cultured and incubated at 28 2°C. The plugs were positioned on either side of the Petri dish's center with 4 cm distance from one another. *R. solani* was inoculated as control. The control's colony size gradually approached that of the plate. All control and test plates were run in triplicate. The following parameters were used to assess pathogen growth inhibition: the percentage of *R. solani* radial growth inhibition at [100 × (*R*1 − *R*2)/*R*1], where *R*1 and *R*2 are the radial growth values of fungi in the control and tested plates, respectively [32]; (ii) the mode of inhibition on a scale of 0 to 4, with 0 denoting no growth inhibition, 1 denoting a growth inhibition of 1 to 25 percent, and 2 denoting a growth inhibition of 26 to 50 percent [33].

### 2.7. Gas Chromatography-Mass Spectrometry (GC/MS) Analysis

GC-MS analysis of ethyl acetate extracts of *Paecilomyces sp.* was performed using a Clarus 500/580 Perkin Elmer GC machine (Connecticut, USA), equipped with an AOC-20i auto-sampler and Elite-1 fused silica capillary column (100 percent methyl polysiloxane, 30 m, 0.25 mm, and 0.25 m), which was used to perform the extraction. As a carrier gas, helium (99.99 percent) was employed at a constant flow rate of 1 ml/min. Samples of 0.5 l were injected with a ratio (1 : 10), while the injector temperature was 280°C. The oven temperature was set to automatically rise from 110°C to 200°C and then at a rate of 5°C/min up to 280°C (10 min). With a runtime of 36 minutes, mass scans were performed in the range of 40–450 Da at electron energy of 70 eV (0.5 sec scan interval). Using the National Institute of Standards and Technology's (NIST) database, the mass spectrum of the GC-MS was analysed. The spectrum of the components kept in the NIST collection was compared to the mass spectrum of the unidentified components [34].

### 2.8. Statistical Analysis

The SPSS software (version 23) was used to assess significant statistical analysis. All the experiments were carried out in triplicates. The one-way ANOVA and the Duncan's test were used to assess whether there were any significant difference between the means.

## 3. Results and Discussion

### 3.1. The Protective Effects of *Pacilomyces sp*. Filtrate on the Growth of *Rhizoctonia solani* on Potato Slices

**Figure 1:**
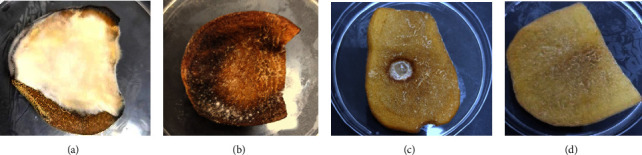
*R. Solani's* reaction to *Paecilomyces* filtrate on potato slices, (a) *R. solani*-inoculated potato slices, (b) solely with PDB medium, (c) with *Paecilomyces* filtrate and *R. solani*, (d) treated with *Paecilomyces* filtrate alone, and. Growth was tested at 28°C for 7 days.

The results showed that potato slices treated with *Paecilomyces* filtrate alone, whether treated with the pathogenic fungus or not, had no clear growth of *R. solani*. The cell-free filtrate inhibited *R. solani* growth on potato slices, recording 100% inhibition at the stock solution concentration (Figures 1(a) and 1(b)). As for the potato slices treated in the PDB medium, whether with or without treatment with the pathogenic fungi, the growth of the *R. solani* appeared clear on the treated slices (Figures 1(c) and 1(d)). This result agrees with that reported previously [30]. An earlier work [31, 35] showed that *Paecilomyces lilacinus* (pt361) cell-free filtrate inhibited the leaf spot caused by *S. sclerotiorum* at all tested concentrations. Many studies have shown that different strains of *Paecilomyces sp*. produced metabolites with antifungal activities, like varioloid A, B [36], paciloxocin A, M [37], monocillin VI, VII [38], aigilomycin B, C, D, LL-2640-1, and 1,2-epoxyaigilomycin D [39]. According to studies, the synthesis of chitinase, leucinotoxins, acetic acid, and protease by *Paecilomyces lilacinus* is the factor responsible for the antinematode activity [40].

### 3.2. Effects of the Filtrate on *Rhizoctonia solani* Growth

**Figure 2:**
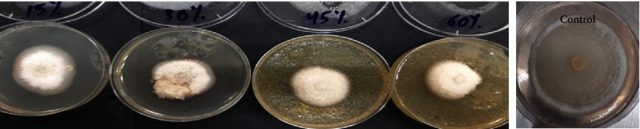
Effects of *Paecilomyces sp*. filtrate on the growth of *R. solani* as tested by the dual agar method on PDA at 25°C for 7 days.

The results showed that *Paecilomyces* filtrate has an effect on the radial growth of the pathogenic *R. solani*, starting from the concentration of 15% to 60%, compared with the control, but at different levels (Figure 2, Table 1). The percentages of radial growth inhibition of pathogenic *R. solani* were 37.5%, 50%, 52.5%, and 56.25% at the concentrations of 15, 30, 45, and 60%, respectively. A previous report [35] found that the cell-free filtrate of *P. lilacinus* (pt361) caused strong inhibition of radial mycelial growth of *S. sclerotium*, ranging from 60.3 to 100%. Another study [31] found that the mycelial growth, germ tube elongation, and spore germination of *Penicillium digitatum* were completely inhibited by the culture filtrate of *Paecilomyces lilacinum* at the concentration of 64%. Diketopiperazine, terezine D secondary metabolites derived from *Paecilomyces cinnamomeus* showed biological activities against *Sordaria fimicola* by their effects on radical growth, causing a 50% reduction at 200 *μ*g/disk [41]. Farinomalein, a maleimide-containing chemical, isolated from *Paecilomyces farinosus* showed strong actions, at 5 g/disk, against the plant *Phytophthora sojae* [42]. Leucinostatin is a complex antibiotic that was isolated from *Paecilomyces lilacinus* [43], of which the compounds leucinostatin A [41] and leucinostatin B [44] were later separated and showed antibacterial and antifungal activities [45]. *Parcilomyces lilacinum* antifungal properties may be attributed to the existence of bioactive metabolites, such as the leucinostatins known as paecilotoxins [46]. Leucinostatins have been shown in several studies to be highly effective against a variety of fungi and bacteria [35, 47, 48].

### 3.3. Test of Antagonistic Activity

**Figure 3:**
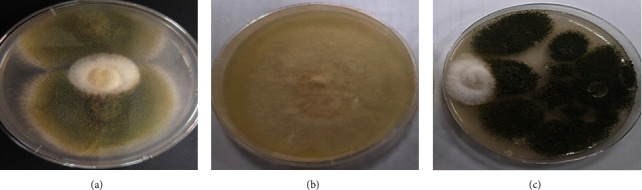
Antagonistic interaction between the endophytic *Paecilomyces sp*. and the pathogenic *Rhizoctonia solani* grown on PDA at 28°C for 7 days.

Dual culture method on the solid medium was performed in the current study to observe the antagonistic nature of the endophytic fungus *Paecilomyces sp.* against the pathogenic fungus *Rhizoctonia solani*. The results listed in Figure 3 show a strong antagonistic impact against the pathogen, where the inhibition rate was 76.25% and on a scale of 4 to determine the level of antagonistic according to [33]. Researchers have indicated that the antagonistic interactions between fungi can be considered as biological control against pathogens [49]. The difference between endophytic fungi and pathogenic indicated the emergence of a specific condition; the endophytic fungus grew above the pathogenic fungus at their point of contact, causing a highly significant inhibition of growth. This case indicates that there has been a mycoparasitism behavior by the endophytic fungus towards the pathogenic one. Researchers [50, 51] have shown that the condition of mycoparasitism is due to the secretion of enzymes by the parasitic fungus, which leads to the decomposition of the walls, and eventually the death, of the partner fungus. It has been found that the cell-free filtrate of *Paecilomyces lilacinus* (pt361) caused a 65% inhibition of radial growth of *Seclerotinia sclerotiorum*, as demonstrated by using the dual culture test, with an inhibition zone of 5.9 mm [35]. Also, it has been reported that, without any physical contact, *Paecilomyces lilacinum* was able to inhibit *Penicillium digitatum* growth by 68.2% based on the dual culture test [31]. It was suggested that this inhibition points to the presence of fungistatic metabolites produced by *Paecilomyces lilacinum* grown on the medium. The production of volatile organic molecules, such as acids, alcohols, alkenes, aldehydes, esters, terpenes, ketones, benzenoids, and pyrazines, as well as the production of bioactive metabolites, enzymes, and toxins, can cause in antagonism relation among the fungi without physical contact. These substances significantly contribute to the antagonistic effects and the fungal recognition systems through chemical signaling [52, 53].

### 3.4. GCMS Analysis

**Figure 4:**
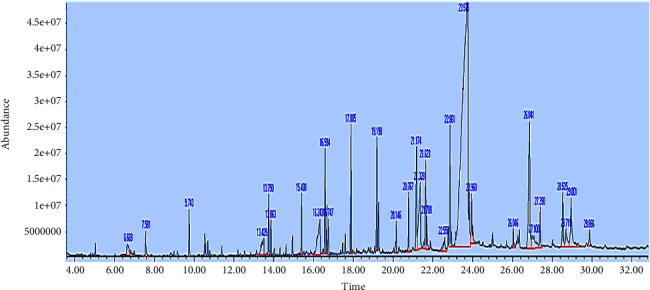
GC-MS chromatograph of crude extracellular ethyl acetate extract of *Paecilomyces sp.*

The ethyl acetate extract of *Paecilomyces sp*. showed a bright yellow color. The composition of the ethyl acetate extracts analyzed by GC-MS is presented in Table 2 and Figure 4. The analysis of *Paecilomyces* extract revealed the presence of of (E) 9, cis-13-Octadecenoic acid, methyl ester (48.607); 1-Heptacosanol, 1-Nonadecene, cyclotetracosane (5.979); 1,2-Benzenedicarboxylic acid, butyl 2-methylpropyl ester, Di-sec-butyl phthalate (3.829); 1-Nonadecene,*n*-Nonadecanol-1, behenic alcohol (3.298); *n*-Heptadecanol-1,1-Hexadecanol, *n*-Pentadecanol (2.962); Dodecanoic acid (2.849); 2,3-dihydroxypropyl ester, Oleic Acid, 9-Octadecenal, (Z)-(2.730).

The major components of the ethyl acetate extract of Paecilomyces were 9, cis-13-Octadecenoic acid, methyl ester, 10-Octadecenoic acid, and methyl ester, which belongs to a group of oleic acid esters with cancer preventive, insectifuge, anti-inflammatory, choleretic, and anemiagenic effects [54]. Due to the presence of many phytoconstituents, including various octadecanoic acid methyl esters, the *Achyranthes ferruginea* plant demonstrated considerable antioxidant, cytotoxic, and free radical scavenging capabilities [55]. Hexadecenoic acid methyl ester had the highest antimicrobial effect against clinical pathogenic bacteria [56]. The fatty acid composition, such as pentadenoic acid (a saturated fatty acids), and showed the antibacterial and antifungal activities of fatty acid methyl esters from the white oak plant extract [57]. Dimethyl phthalate may cause membrane channel misopening and cell membrane deformation. Isolated from *B. mcbrellneri*, di (2-ethylhexyl) phthalate and di-n-butyl phthalate exhibit a broad-spectrum of antibacterial activities [58]. Gram-positive *S. epidermidis* and *S. aureus* and Gram-negative *E. coli*, *P. aeruginosa*, and *Klebsiella pneumoniae* are all susceptible to being inhibited by phthalates. Di (2-ethylhexyl) phthalate from *Calotropis gigantean* flowers has antibacterial effects on *B. subtilis* [59] and antifungal activities against *Candida albicans* [60]. The chemical di-n-butyl phthalate, obtained from a new marine Streptomyces species [61], greatly reduced the spore germination and mycelial growth of *Colletotrichum fragariae*. Di-n-butyl phthalate was reported to inhibit mycelium growth and spore germination of *Colletotrichum musae*, *Gaeumannomyces graminis*, and *Colletotrichum gloeosporioides* [62, 63]. Long-chain primary alcohols are present in the *S. amplexicaulis* leaf extract. A crude extract can be made from the leaves of this plant, which is a synthetic mixture of six chemicals (1-tridecanol, 1-pentadecanol, 1-heptadecanol, 1-nonadecanol, 1-eicosanol, and 1-tricosanol). Lower amounts of *S. amplexicaulis* leaves demonstrated antibacterial action [64]. Cyclotetracosane (hydrocarbon) possesses higher biological activities than the other fractions of *Jatropha zeyheri* [65].

## 4. Conclusions

*Paecilomyces sp.* is a type of endophytic fungi that plays an important role in biological control. GC-mass fungus extract results show different types of secondary metabolites that possess antifungal and antibacterial properties. Antagonistic interactions between fungi can be considered as biological control against pathogens. The use of biologically active secondary metabolites of endophytic fungi as a biological control against plant diseases is essential and will help mitigate the harmful side effects of the use of synthetic pesticides on plant growth and crop production. Thus, the production of pesticides from the secondary metabolites of endophytic fungi will go a long way towards improving food security.

## Figures and Tables

**Table 1:** Effects of the filtrate on *Rhizoctonia solani* growth.

Concentration (%)	Colony diameter(mm) mean ± SD
15	5.06 ± 0.05^b^
35	4.06 ± 0.06^b^
45	3.83 ± 0.05^c^
60	3.53 ± 0.05^c^
Control	8.06 ± 0.06^a^

^*∗*^Different letters represented significant difference at *P* ≤ 0.05.

**Table 2:** Bioactive compounds identified in the ethyl acetate extract of *Paecilomyces sp.* Data from NIST standard reference database: *NIST chemistry webbook*.

Peak	Library ID	Area %	R. Time
1	Acetic acid, 2-ethylhexyl ester	0.988	9.743
2	*n*-Decanoic acid	1.396	13.429
3	1-Tetradecanol, Trichloroacetic acid, *n*-Tridecan-1-ol	1.083	13.750
4	Tetradecane	0.567	13.863
5	Phenol, 2,5-bis (1,1-dimethylethyl), Oxirane, [[4-(1,1-dimethylethyl) phenoxy]methyl]-	1.023	15.400
6	Dodecanoic acid	2.849	16.243
7	n-Heptadecanol-1, 1-Hexadecanol, *n*-Pentadecanol	2.962	16.594
8	Fluoroacetic acid, dodecyl ester, Lauryl acetate	0.623	16.747
9	2-Propenoic acid, tetradecyl ester, 2-Propenoic acid, pentadecyl ester, 2-Propenoic acid, tridecyl ester	2.656	17.885
10	1-Nonadecene, *n*-Nonadecanol-1, Behenic alcohol	3.298	19.198
11	8-Pentadecanone, 5-Keto-2,2-dimethylheptanimine, Decanoic acid, 2-propenyl ester	1.255	20.146
12	Hexadecanoic acid, methyl ester	3.040	21.174
13	Butyl 2-methylpropyl ester, di-butyl phthalate, 1,2-Benzenedicarboxylic acid, di-sec-butyl phthalate	3.829	21.328
14	n-Hexadecanoic acid, Pentadecanoic acid	2.468	21.623
15	1-Nonadecene Behenic alcohol, 1-Heneicosanol	0.660	21.708
16	Heptacosane, 1-chloro-, Tritetracontane, Tetratetracontane	0.800	22.558
17	10-Nonadecanone	2.976	22.861
18	Octadecenoic acid, methyl ester, (E) cis-13-Octadecenoic acid, methyl ester, 9-Octadecenoic acid, methyl ester	48.607	23.575
19	9-Octadecenoic acid, Oleic acid, 6-Octadecenoic acid	1.698	23.960
20	1-Nonadecene, Behenic alcohol, 1-Heneicosanol	0.552	26.046
21	1-Heptacosanol, 1-Nonadecene, Cyclotetracosane	5.979	26.841
22	Cyclohexanecarboxylic acid, pentadecyl ester, Cyclohexanecarboxylic acid, undecyl ester, 2-Isobutoxy-4-methyl-[1–3] dioxaborinane	1.524	27.100
23	1-Cyano-4-(5-hexenyl)benzene, cis-11-Hexadecenal, 13-Octadecenal, (Z)-	1.524	27.100
24	Phthalic acid, di (2-propylpentyl ester, Bis (2-ethylhexyl) phthalate	0.934	27.390
25	3-n-Butylthiophene-1,1-dioxide, difluoro (methylamino) phosphine sulfide, 1,3-Dioxane-2-ethanol, tert-butyldimethylsilyl ether of 5,5-dimethyl dioxane	1.688	28.525
26	Benzenepropanoic acid, 4-(1H-1,2,3,4-tetrazol-1-yl)-, cis, 6-Octadecenoic acid, trimethy lsilyl ester, 5,5-dimethyl-1,3-dioxane-2-ethanol, tert-butyldimethylsilyl ether	1.070	28.719
27	Oleic acid, 2,3-dihydroxypropyl ester, and 9-Octadecenoic acid (Z) the ninth decade	2.730	29.001
28	Hexanoic acid, pentadecyl ester Hexanoic acid, hexadecyl ester, 2-Ethylbutyric acid, nonyl ester	0.581	29.800

## Data Availability

All data in this study are included within the article.
